# Item non-response imputation in the Korea National Health and Nutrition Examination Survey

**DOI:** 10.4178/epih.e2022096

**Published:** 2022-10-28

**Authors:** Serhim Son, Hyemi Moon, Hyonggin An

**Affiliations:** Department of Biostatistics, Korea University College of Medicine, Seoul, Korea

**Keywords:** Korea National Health and Nutrition Examination Survey, Methods, Sample size, Database

## Abstract

**OBJECTIVES:**

The Korea National Health and Nutrition Examination Survey (KNHANES) is a public health survey that assesses individuals’ health and nutritional status and monitors the prevalence of major chronic diseases. In general, sampling weights are adjusted for unit non-responses and imputation is conducted for item non-responses. In this study, we proposed strategies for imputing item non-responses in the KNHANES in order to improve the usefulness of data, minimize bias, and increase statistical power.

**METHODS:**

After applying logical imputation, we adopted 2 separate imputation methods for each variable type: unweighted sequential hot-deck imputation for categorical variables and sequential regression imputation for continuous variables. For variance estimation, multiple imputations were applied to the continuous variables. To evaluate the performance of the proposed strategies, we compared the marginal distributions of imputed variables and the results of multivariable regression analysis for the complete-case data and the expanded data with imputed values, respectively.

**RESULTS:**

When comparing the marginal distributions, most non-responses were imputed. The multivariable regression coefficients presented similar estimates; however, the standard errors decreased, resulting in statistically significant p-values. The proposed imputation strategies may cope with the loss of precision due to missing data, thus enhancing statistical power in analyses of the KNHANES by providing expanded data with imputed values.

**CONCLUSIONS:**

The proposed imputation strategy may enhance the utility of data by increasing the number of complete cases and reducing the bias in the analysis, thus laying a foundation to cope with the occurrence of item non-responses in further surveys.

## GRAPHICAL ABSTRACT

[Fig f3-epih-44-e2022096]

## INTRODUCTION

Public health is a topic of major concern. Most health institutions, therefore, conduct health-related surveys and utilize them in individual health-centered studies. The Korea National Health and Nutrition Examination Survey (KNHANES), implemented by the Korea Disease Control and Prevention Agency, is a nationwide cross-sectional annual survey conducted among a cohort of approximately 10,000 individuals. This survey assesses individual health and nutritional status, and monitors the prevalence of major chronic diseases such as hypertension, diabetes, and cancer, to frame new or reform existing health policies [1]. The KNHANES includes health interviews, health examinations, and nutrition surveys. The health interviews collect information such as demographics, smoking and drinking behaviors, and comorbidities. The health examinations include oral and eye tests, otorhinolaryngology, dual-energy X-ray absorptiometry (DXA), and clinical laboratory assessments. The nutrition surveys include questions on food intake status and dietary behavior. To ensure data accuracy and diversity, this survey has been designed by statistical experts and includes verified questionnaires that are administered by skilled interviewers [2]. Despite these efforts, it is challenging to maintain data precision due to missing values that mostly stem from a lack of responses. Instances of non-response may occur when respondents refuse to answer questions directed towards sensitive or personal details such as income, when respondents are simply unaware of the answer to a specific question, or when interviewers fail to ask a question or note down the answer [3].

To avoid the problems posed by non-response data, complete-case analysis is used to exclude respondents who have provided incomplete information. However, this method decreases the sample size, and may, therefore, cause a loss of statistical power and precision. In addition, systematic differences can exist between respondents and non-respondents [4]. Owing to irregularities in data patterns and difficulties in using the standard software, data analysis and handling can become complicated [5].

Survey non-responses include 2 basic types: unit non-responses and item non-responses. A unit non-response is defined as the failure of an individual to respond to the survey, whereas an item non-response refers to the failure of an individual to respond to a particular survey question. In general, unit non-responses are adjusted using weights and item non-responses are adjusted using imputation. Imputation is a method of filling in missing data with plausible values using a statistical model. Missing data imputation enables users to conduct a general statistical analysis without loss of information. However, if the imputation model is incorrect, the estimates obtained from an analysis using filled data can cause serious bias. Therefore, multiple factors, including the type, characteristics, and cause of missing data, must be considered.

In the United States, the third National Health and Nutrition Examination Survey, conducted by the Centers for Disease Control and Prevention, incorporated a multivariate linear random-effects statistical model for data analysis and used a Markov-chain Monte Carlo (MCMC) algorithm to perform 5 sets of imputations to fill the response gaps [6]. The Household Longitudinal Study in the United Kingdom used predictive mean matching and hot deck imputation for a cross-sectional study, and a multiplicative model for a longitudinal study [7]. Basic research on occupational safety and health panel studies in Korea uses highly correlated variables based on financial status. An imputation method assuming a multivariate distribution should therefore be typically applied to such studies. However, owing to application difficulties associated with this technique, convenient methods such as hot decks, ratio estimation, and stochastic imputation are used [8].

The purpose of our study was to provide data with imputed values for item non-responses that occurred in the fifth and sixth KNHANES. This was performed to obviate the need for user-determined imputations for individual studies. First, we imputed item non-responses using logical, non-parametric, and parametric imputation methods. We then compared the marginal distributions of the variables with a high non-response rate and multivariable regression using imputed data. Our data imputation provides a straightforward way for other users to easily interpret, analyze, and obtain results with less bias and high precision.

## MATERIALS AND METHODS

### Selection of variables for imputation

The variables for imputation were selected as follows. First, we conducted a general inspection of non-responses while assessing the non-response rate. For example, while the non-response rate was relatively low for the interview-type queries, self-reported questions such as smoking and drinking had higher non-response rates. In addition, for smoking-related questions, there were more cases where all variables were unanswered in a section than where multiple related variables were unanswered. Based on the inspection of non-responses, non-responses for each variable were defined as item non-responses or unit non-responses.

To select the imputation variables, we designated 7 datasets in the KNHANES as follows: basic data (ALL), injury and medical use data (IJMT), oral data (OE), DXA, eye data (EYE), ear-nose-throat data (ENT), and food frequency questionnaire (FFQ). ALL contained the largest amount of combined information on demographics, chronic diseases, and laboratory data. IJMT consisted of questions about hospitalization and medical services. OE was based on information about the respondent’s oral status and health. DXA was related to body mineral density, bone mineral content, and body fat percentage. EYE included questions about eye conditions, such as vision, cataracts, and glaucoma, while ENT comprised conditions of the ear, nose, and throat, such as rhinitis and inflammation in the ear. FFQ was directed toward the respondents’ diet, including the type, frequency, and intake of food.

The detailed process for each dataset was as follows: common to all data, individuals who did not participate in the health examination were excluded from most of the questions except for a few. ALL included all variables, except the nutrition survey. IJMT included questions about whether respondents used medical services, and the frequency of use was selected as the imputation variable. The number of vaccinations in ALL and the impairment and hospitalization, outpatient, and pharmacy uses in the IJMT were excluded owing to their low frequency and difficulty in considering the joint distribution. Questions on oral health, dental pain, and orthodontic treatment experiences were included in the OE data. Although these questions were a part of the examination, they were selected as variables for imputation, as they were factors involving self-diagnosis. While the DXA was an examination question, the participants were identifiable using the variable “examination respondents”; therefore, logical imputation was possible. In addition, all interview questions also existed, and all questions were selected as variables for imputation in the DXA data. EYE and ENT were excluded from the imputation variables because the conditions under which the subjects underwent the examination were unclear, and most of the non-responses were a result of non-examination. The nutrition survey data were excluded owing to difficulties in imputation because respondents were investigated more than once, and hence had more than 1 row. Additionally, newly defined variables using raw data were excluded.

### Number of selected non-response questions and item non-response rates in the Korea National Health and Nutrition Examination Survey

Before imputation, the number of questions with non-responses was quantified. The distribution of non-response questions in 2010 is shown in Table 1. These values were calculated after logical imputation. The details of the logical imputation are presented in Logical Imputation. Out of 649 questions that were selected for imputation, 560 had non-responses, most of which were part of ALL (Table 1). The non-response rates (i.e., the proportion of questions for which non-responses existed) were 84.01%, 91.81%, 92.05%, 92.80%, and 91.25% in 2011, 2012, 2013, 2014, and 2015, respectively.

### Logical imputation

Logical imputation is a method of using logical conditions to answer a question; thus, with logical imputation, non-response values can be uniquely determined within the relevant data [9]. The logical imputation was divided into 3 parts. The first part comprised household questions. In the health interviews, household questions included items such as the number of household members, composition codes, and income. If the respondents had the same household ID, the data must have the same value. If there was a non-response to a household question, the household ID was checked and imputed with the same value.

Based on the health examination, respondents were classified according to an indicator variable named “examination respondents.” Data for non-responders who participated in the health examination were corrected to “not applicable,” “don’t know/non-response,” or “unscreened” according to the age condition. Those who did not correspond to the age condition were imputed as “not applicable,” owing to their ineligibility, while those who responded to the age condition but did not have any values were imputed as “don’t know/non-response” or “unscreened.” For individuals who did not participate in the health examination, the lack of values was reasonable; therefore, the non-response values were retained. Appropriate examples are listed in Supplementary Material 1. Considering the variable “height” in KNHANES 2010 as an example, without accounting for the variable “examination respondents,” a total of 8,424 response and 534 non-response values were available. However, upon accounting for the variable “examination respondents,” for the age condition of 1 year or older, 8,466 individuals were eligible for the health examination, and data for 42 individuals were found to be missing. Therefore, logical imputation was performed to correct values from missing to “don’t know/non-response” for all 42 people.

In addition, there were filter questions in the KNHANES. For example, 1 of the questions included whether respondents brush their teeth or use oral health products. Individuals who answered “yes” were qualified to answer follow-up questions. For some data collected from 2011, when the response value of a follow-up question existed, but the response value of the filter question was absent, logical imputation was performed.

### Imputation method

The KNHANES includes examination surveys; therefore, participants are often unscreened for specific tests. In our study, unscreened individuals were defined as unit non-responses. In addition, unit non-responses also occurred when respondents did not answer all the questions in the health surveys. Except for unit non-responses, the other non-responses were defined as item non-responses. To impute item non-responses, 2 methods were used depending on the type of variable. For categorical variables, unweighted sequential hot-deck imputation was used, and for continuous variables, sequential regression imputation was performed. For unweighted sequential hot deck imputation, SAS macros were used [10,11]. Sequential regression imputation was conducted using IVEware software (Survey Research Center, Institute for Social Research, University of Michigan, Ann Arbor, MI, USA) [12].

#### Unweighted sequential hot deck imputation

Hot deck imputation uses the values of other respondents to replace missing values. Respondents who provide values are referred to as donors, and respondents for whom the data are replaced by the provided values are referred to as donees. Hot deck imputation includes methods such as the nearest-neighbor hot deck and sequential hot deck, depending on the algorithm used for donor selection. To conduct hot-deck imputation, we used auxiliary variables. Auxiliary variables are those that are not included in the analysis but can enhance the precision of the imputation. These include class, sorting, and stratification variables. Class and sorting variables were used as stratification variables to identify the nearest neighbor values in the same group for imputation.

The KNHANES involves a 2-stage stratified cluster sampling design, which should be considered when using the data. Andridge & Little [13] showed that the use of sampling design variables as stratifying variables provided the most robust results. Unweighted sequential hot deck imputation stratifies all respondents into homogeneous strata using class variables, sorts respondents using variables related to the donees’ tendencies (e.g., age and income), and selects adjacent observations as donors. In each stratum, the serpentine sorting algorithm is recommended, as it ensures that selected adjacent observations are as similar or connected as possible [10]. This algorithm sorts the variables in ascending and descending order sequentially, so that the adjacent values are similar. In this study, we used 10 auxiliary variables (age, personal income, family income, and education levels) as sorting variables, and strata for variance estimation, primary sampling units, region, town, apartment/non-apartment, and sex as class variables.

For categorical variables, multiple imputations may confuse the users. For example, a respondent could be imputed as “1. Yes,” for marital status for the first imputation. However, for the second imputation, the respondent could be imputed as “0. No.” To address this confusion, single imputation was performed.

#### Sequential regression imputation

Sequential regression imputation can be used to impute missing data by setting parametric models that include various types of variables, restrictions, and bounds. Logistic regression can be used for binary variables, regression models for continuous variables with a normal distribution, and Poisson regression models for count variables [14].

Assuming that *Y*_1_, *Y*_2_, …, *Y_p_* denote the *p* variables with missing values, the joint conditional distribution density of *Y*_1_, *Y*_2_, …, *Y_p_* can be expressed as,

f(*Y*_1_, *Y*_2_, …, *Y_p_*|*X*, *θ*_1_, *θ*_2_, …, *θ_p_*)=*f*_1_(*Y*_1_|*X*, *θ*_1_)*f*_2_(*Y*_2_|*X*, *Y*_1_, *θ*_2_) … *f_p_*(*Y_p_*|*X*, *Y*_1_, *Y*_2_, …, *Y*_*p*-1_, *θ_p_*)

where *X* is the predictor matrix and *θ_i_*, *i*=1, …, *p* is a vector of parameters in the conditional distribution. Instead of a conditional distribution, *θ_i_* can be drawn from f(*Y_j_*|*X*,*Y*_1_^(*c*)^, …, *Y*_*j*−1_^(*c*)^, *Y*_*j*+1_^(*c*)^, …, *Y_p_*^(*c*)^, *θ_p_*), *j*=1, … *p* using MCMC. Imputation is then performed by repeating the process of drawing the missing data *Y_mis_* under the observed data, and *Y_obs_* and *θ_p_* are known.

Continuous variables were imputed using the same auxiliary variables as categorical variables. However, unlike categorical variables, single imputation does not reflect the uncertainty of non-response values, and the variance of an estimate can be underestimated [15,16]. To consider uncertainty, resampling methods, such as bootstrap and jackknife, uncertainty is estimated and added to the variance estimation. Multiple imputation is another approach that can impute the same value several times [17,18]. After several imputations, the results are combined and a single estimate is derived [15]. In this study, as the imputed data are for public use, 5 multiple imputations were performed.

### Ethics statement

The KNHANES is a survey implemented directly by the nation for public welfare, and is conducted without IRB consideration.

## RESULTS

### Descriptive statistics

Among the imputation variables, those with high non-response rates and remarkable changes from 2010 to 2015 were presented as results. As our study had no true values, we compared the descriptive statistics before and after imputation [19]. For continuous variables, the number (n), mean, standard deviation, and median values were presented. After 5 multiple imputations, the results were combined, and the mean and standard error were presented. For categorical variables, the values were presented as frequencies and percentages.

**Figure 1.**
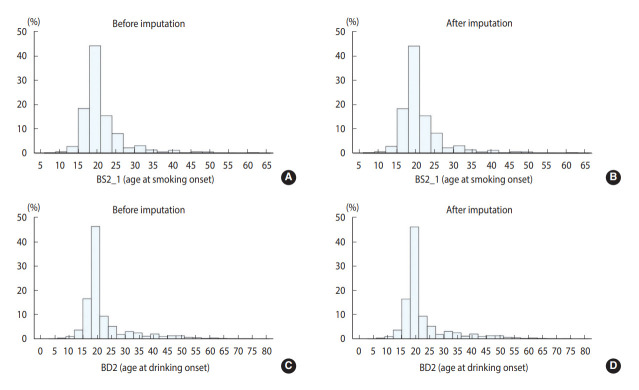
Results of item non-response imputation for continuous variables. BS2_1 (age at smoking onset), (A) before imputation and (B) after imputation. BD2 (age at drinking onset), (C) before imputation and (D) after imputation.

**Figure 2.**
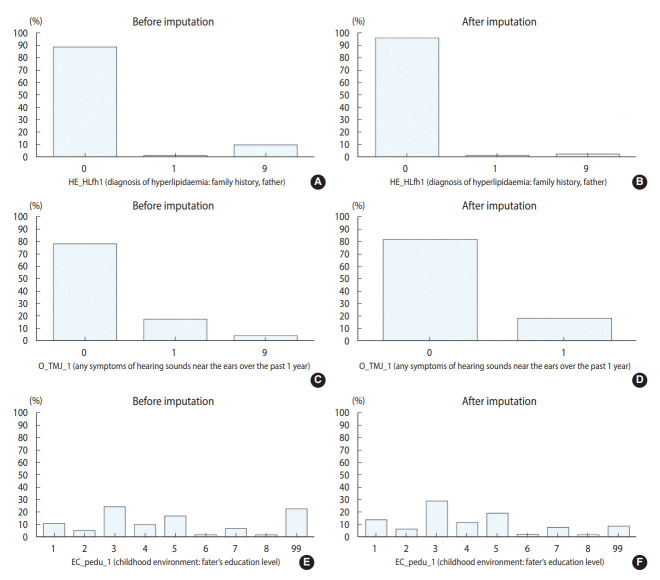
Results of item non-response imputation for categorical variables. HE_HLfh1 (diagnosis of hyperlipidaemia: family history, father), (A) before imputation and (B) after imputation. O_TMJ_1 (any symptoms of hearing sounds near the ears over the past 1 year), (C) before imputation and (D) after imputation. EC_pedu_1 (childhood environment: father’s education level), (E) before imputation and (F) after imputation.

Tables 2 and 3 show the results for the continuous and categorical variables, respectively. For variable “BS2_1,” which queried about the age at smoking onset, 11 item non-responses were imputed, and there were no noticeable differences between raw and imputed data. The variable “BD2” included questions on the age at drinking onset. Before imputation, the average age at onset was 22.54 years. Through imputation, 19 item non-responses were imputed, presenting similar descriptive statistics. A histogram (Figure 1) shows comparable distributions among Figure 1A-D. For categorical variables, most of non-responses were imputed. Among the variables concerned with the father’s family history of hyperlipidemia, 502 respondents with “don’t know/non-response” were imputed. As data for a considerable number of respondents were imputed, the change was conspicuous in Figure 2A and B. In total, 496 respondents were imputed as “0. No” and six respondents were imputed as “1. Yes.” After imputation, the non-response rate decreased to 2.53%. Considering the variables in OE, “O_TMJ_1” included questions about symptoms of hearing sounds close to the ears over the past 1 year. There were 284 non-responses, and after imputation, all non-responses were imputed as either “0. No” or “1. Yes.” As shown in Figure 2C and D, all values of “9. Don’t know” were imputed as either “0. No” or “1. Yes.” Lastly, for “EC_pedu_1,” with questions about the father’s education level, among the 1,259 respondents with “99. Don’t know/non-response,” 773 values were imputed, and this variable showed one of the most noticeable changes, as presented in Figure 2E and F. Most of the “99. Don’t know/non-response” values were imputed as “3. Under elementary school.” After imputation, the non-response rate decreased to 8.72%.

### Multivariable regression using imputed data

Multivariable regression analysis was performed using imputed data for 2015. We used “age at first childbirth” as the dependent variable, and “father’s education level,” “mother’s education level,” “types of health insurance,” “private medical insurance,” “age at drinking onset,” and “education level” as predictors. Before imputation, 1,891 respondents were included in the complete case dataset. Five multiple imputations were performed, and the obtained estimates were combined. After imputation, the number of completed cases increased to 2,434. The results of multivariate regression are listed in Table 4. Before imputation, at a 5% significance level, only “education level” was significant (p<0.01). After imputation, “mother’s education level,” “private medical insurance,” and “education level” were significant (p<0.01, < 0.01, and < 0.01, respectively). There were no substantial changes in the coefficient value; however, the significance level differed. This may have been because after using the imputed dataset, the standard error decreased, but the coefficients remained similar; therefore, the test statistics increased and the p-value decreased. A decreased standard error also indicated an increased statistical power overall, implying the necessity of imputation.

## DISCUSSION

The primary goal of this study was to provide item non-response imputed data from the fifth and sixth KNHANES and enable researchers to utilize the data for analysis. Through this study, we presented the process of data imputation and its effect on the statistical power of analysis. When data analysis was performed after imputation, the standard error decreased, resulting in an increment of statistical power. Although the imputed data have not yet been made accessible, we believe that once published, they will make it possible for users to easily obtain results with minimum bias and high-precision.

Among the various issues related to multiple imputations, several studies have suggested the proper number of multiple imputations. In our study, we performed 5 imputations; as the KNHANES is aimed to benefit researchers in terms of data analysis, an increase in the amount of imputed data may be difficult for users to interpret and analyze. However, in the process of imputation, the number of imputations is also an important factor to consider. Bodner [20] showed that as the missing rate increased, the number of imputations also increased. Bodner suggested that the number of imputations should be equal to or greater than the missing rate. Graham et al. [21] also showed that if the missing rate is 10%, an approximately 4% loss of power occurs when using fewer than 10 imputations as compared to when using 100 imputations. However, if the missing rate increases to 70%, the loss of power is 22% under the same imputation conditions. Similar results were obtained in terms of the statistical power and standard error variability. With a missing rate of 76%, 20 imputations were highly appropriate for decreasing variability in the standard error [22]. When the missing rate is high, the data collection process must be reviewed prior to analysis. Nevertheless, if the missing rate continues to be high, imputation can be used to create imputed data for analysis.

Some limitations of this study should be noted. Even though we included as many common auxiliary variables in the imputation model as possible, we could consider some more auxiliary variables throughout the entire survey. In general, when constructing imputation models, it is recommended to include as many predictors as possible [23-25]. This is called the principle of congeniality, which remains valid even if the predictor contains noise [24]. As there are many data users, the purpose of using the data varies; therefore, the imputed data should be as general as possible when using the saturated model. Therefore, in addition to the auxiliary variables considered in this study, it was possible to select and include more relevant variables in the imputation model. The other challenge relates to missing assumptions. The missing at random (MAR) assumption means that non-responses are related to the observed data. However, non-responses can depend on missing data themselves, even after we consider the observed data. This is called the missing not at random assumption. Since these are assumptions involved in non-responses, they cannot be verified from observed data. Therefore, a sensitivity analysis to examine the correctness of the MAR assumption could be another topic for further research [26].

To summarize, the occurrence of non-responses is inevitable in surveys. Reducing non-responses is desirable; however, if this fails, the data quality can be improved by correcting non-responses through post-processing. Although imputation fills the non-response values, it is noteworthy that the purpose of imputation is not to obtain real values, but rather plausible values using information known in advance to achieve the best estimate [18]. Through this study, the validity of statistics using imputed data can be improved, and imputed data can be disclosed to users for convenient use. In addition, the investigation of non-responses can be useful for formulating strategies to improve response rates in future data.

## Figures and Tables

**Figure f3-epih-44-e2022096:**
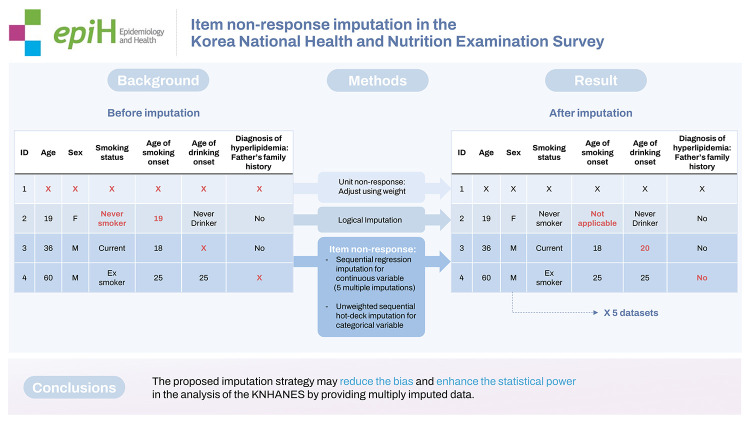


**Table 1.** Numbers and rates of item non-response questions in the fifth KNHANES (2010)

Dataset	No. of respondents	No. of questions selected for imputation	No. of non-response questions (%)
ALL	8,958	534	509 (95.32)
IJMT	8,473	8	8 (100)
OE	8,473	2	2 (100)
DXA	7,043	105	41 (39.05)
Total	-	649	560 (86.29)

KNHANES, Korea National Health and Nutrition Examination Survey; ALL, basic data; IJMT, injury and medical use data; OE, oral data; DXA, dual-energy X-ray absorptiometry.

**Table 2.** Results of item non-response imputation (continuous variables)

Variables	n	Mean±SD	Median
Age at smoking onset (BS2_1) – the fifth KNHANES (2012)			
Before imputation	2,175	20.95±6.30	20.00
After imputation			
First MI	2,186	20.96±6.30	20.00
Second MI	2,186	20.95±6.29	20.00
Third MI	2,186	20.95±6.30	20.00
Fourth MI	2,186	20.95±6.30	20.00
Fifth MI	2,186	20.95±6.31	20.00
Combined mean±SE^1^		20.95±0.13	
Age at drinking onset (BD2) – the sixth KNHANES (2015)			
Before imputation	4,867	22.54±9.76	20.00
After imputation			
First MI	4,886	22.57±9.76	20.00
Second MI	4,886	22.57±9.77	20.00
Third MI	4,886	22.57±9.77	20.00
Fourth MI	4,886	22.57±9.77	20.00
Fifth MI	4,886	22.57±9.77	20.00
Combined mean±SE^1^		22.57±0.31	

SD, standard deviation; KNHANES, Korea National Health and Nutrition Examination Survey; MI, multiple imputation; SE, standard error.

1 ”Combined” denotes the combined results of 5 multiple imputations.

**Table 3.** Results of item non-response imputation (categorical variables)

Variables^1^	Imputation	
	Before	After
Diagnosis of hyperlipidemia: Father’s family history (HE_HLfh1) - the fifth KNHANES (2012)		
0. No	6,022 (88.61)	6,518 (95.91)
1. Yes	100 (1.47)	106 (1.56)
9. Don’t know/non-response	674 (9.92)	172 (2.53)
Symptoms of hearing sounds close to the ears, over the past 1 year (O_TMJ_1) - the fifth KNHANES (2012)		
0. No	5,176 (78.19)	5,406 (81.66)
1. Yes	1,160 (17.52)	1,214 (18.34)
9. Don’t know	284 (4.29)	-
Childhood environment: Father’s education level (EC_pedu_1) – the sixth KNHANES (2015)		
1. Uneducated	606 (10.88)	767 (13.77)
2. Korean traditional school	287 (5.15)	355 (6.37)
3. Under elementary school	1,351 (24.25)	1,611 (28.92)
4. Under middle school	554 (9.94)	642 (11.52)
5. Under high school	941 (16.89)	1,063 (19.08)
6. Under college	96 (1.72)	114 (2.05)
7. Under university	384 (6.89)	430 (7.72)
8. Graduate school or higher	93 (1.67)	103 (1.85)
99. Don’t know/non-response	1,259 (22.60)	486 (8.72)

Values are presented as frequency (%). KNHANES, Korea National Health and Nutrition Examination Survey; HE_HLfh, diagnosis of hyperlipidaemia: family history, father; O_TMJ_1, any symptoms of hearing sounds near the ears over the past 1 year; EC_pedu_1, childhood environment: father’s education level.

1 Numbers are added to indicate that it is the same variable as the KNHANES guideline.

**Table 4.** Comparison of multivariable regression before and after imputation

Variables^1^		Before imputation					After imputation				
		Coef	SE	95% CI		p-value	Coef	SE	95% CI		p-value
				LL	UL				LL	UL	
Intercept		18.07	0.42	17.24	18.89	<0.01	19.10	2.13	14.57	23.63	<0.01
Father’s education level						0.95					0.80
	1. Uneducated			Reference					Reference		
	2. Korean traditional school	-0.38	0.46	-1.29	0.53	0.41	-0.03	0.41	-0.84	0.78	0.94
	3. Under elementary school	-0.02	0.35	-0.71	0.68	0.96	0.15	0.29	-0.42	0.72	0.61
	4. Under middle school	0.12	0.41	-0.69	0.92	0.77	0.24	0.34	-0.42	0.91	0.47
	5. Under high school	0.08	0.45	-0.82	0.97	0.87	0.20	0.36	-0.51	0.91	0.59
	6. Under college	-0.46	0.71	-1.87	0.95	0.52	-0.63	0.60	-1.80	0.55	0.30
	7. Under university	0.28	0.59	-0.88	1.45	0.63	-0.03	0.49	-0.99	0.93	0.95
	8. Graduate school or higher	0.21	0.87	-1.50	1.91	0.81	0.34	0.65	-0.94	1.61	0.60
Mother’s education level						0.06					<0.01
	1. Uneducated			Reference					Reference		
	2. Korean traditional school	-0.06	0.60	-1.24	1.13	0.92	-0.09	0.52	-1.12	0.93	0.86
	3. Under elementary school	0.34	0.30	-0.26	0.93	0.27	0.26	0.25	-0.22	0.74	0.28
	4. Under middle school	0.71	0.42	-0.11	1.53	0.09	0.92	0.33	0.27	1.58	0.01
	5. Under high school	0.61	0.44	-0.25	1.47	0.16	0.95	0.38	0.21	1.70	0.01
	6. Under college	1.42	1.44	-1.42	4.26	0.32	2.07	1.39	-0.66	4.81	0.14
	7. Under university	0.36	0.66	-0.94	1.65	0.59	1.51	0.89	-0.24	3.25	0.09
	8. Graduate school or higher	-1.72	0.92	-3.55	0.10	0.06	-1.88	0.78	-3.42	-0.35	0.02
Private medical insurance						0.11					0.01
	1. Yes	0.45	0.28	-0.11	1.00	0.11	0.61	0.22	0.15	1.06	0.01
	2. No			Reference					Reference		
Family income		0.00	0.00	0.00	0.00	0.79	0.00	0.00	0.00	0.00	0.27
Age at drinking onset		0.00	0.00	0.00	0.00	0.06	0.00	0.00	0.00	0.00	0.41
Education level						<0.01					<0.01
	1. Uneducated			Reference					Reference		
	2. Korean traditional school	4.17	0.58	3.03	5.31	<0.01	2.63	2.10	-1.84	7.10	0.23
	3. Under elementary school	4.53	0.49	3.57	5.50	<0.01	3.46	2.13	-1.09	8.01	0.13
	4. Under middle school	5.56	0.53	4.51	6.61	<0.01	4.29	2.09	-0.22	8.79	0.06
	5. Under high school	6.77	0.53	5.73	7.82	<0.01	5.63	2.13	1.06	10.20	0.02
	6. Under college	7.91	0.59	6.74	9.08	<0.01	6.53	2.15	1.95	11.11	0.01
	7. Under university	9.18	0.57	8.06	10.29	<0.01	7.88	2.13	3.33	12.44	<0.01
	8. Graduate school or higher	9.93	0.65	8.65	11.20	<0.01	8.74	2.16	4.13	13.34	<0.01

Coef, coefficient; SE, standard error; CI, confidence interval; LL, lower limit; UL, upper limit.

1 Numbers are added to indicate that it is the same variable as the KNHANES guideline.

## SUPPLEMENTARY MATERIALS

Supplementary Material 1. Logical imputation
