# Supplementary material for: Item non-response imputation in the Korea National Health and Nutrition Examination Survey
Source: Epidemiol Health. 2022 Oct 28;44:e2022096. doi: 10.4178/epih.e2022096 (PMC10106541; doi:10.4178/epih.e2022096)
Supplement: Supplementary Material 1. — Logical imputation [file epih-44-e2022096-Supplementary-1.docx]

Supplementary Material 1. Logical imputation

| **Examination respondents** | **ID** | **Age** | **Age conditions** | **True value** | **After logical imputation** |
| --- | --- | --- | --- | --- | --- |
| o | 1 | 5 | x | . | Non-applicable |
| o | 2 | 17 | x | 100 | Non-applicable |
| o | 3 | 21 | o | 121 | 121 |
| o | 4 | 40 | o | . | Unscreened or don’t know/non-response |
| x | 5 | 58 | . | . | . |

Supplementary Material 1 shows an example of logical imputation. As ID 5 does not include an ‘examination respondents’, logical imputation is not performed, and the non-response value is left as blank. When the age condition is 19 years or older, ID 1 and ID 2 do not satisfy the age condition; therefore, they are logically imputed as ‘non-applicable’. In the case of ID 3 and ID 4, they include an ‘examination respondents’ and satisfy the age condition; therefore, the result of logical imputation is ‘don’t know/non-response’ and ‘unscreened’. Because ID 3 was examined as 121, logical imputation is not performed. In contrast, in the case of ID 4, as the value was not examined, it is imputed as ‘unscreened’ or ‘don’t know/non-response’.
